# Modeling the Pathways of Knowledge Management Towards Social and Economic Outcomes of Health Organizations

**DOI:** 10.3390/ijerph16071114

**Published:** 2019-03-28

**Authors:** Ion Popa, Simona Cătălina Ștefan

**Affiliations:** Department of Management, The Bucharest University of Economic Studies, Bucharest 010374, Romania; ion.popa@man.ase.ro

**Keywords:** economic outcomes, knowledge management process, healthcare, PLS-SEM, quality of care, social outcomes

## Abstract

Despite the increasing emphasis placed on knowledge management (KM) by the business sector and the common belief that creating, acquiring, sharing, and the use of knowledge enable individuals, teams, and communities to achieve superior performance, within the healthcare context, there is still room from improvements from both the theoretical and empirical perspectives. The purpose of this paper is to outline the contribution of KM process to the social- and economic-related outcomes in the context of health organizations. Given the theoretical approach on the considered concepts and their relationships, a conceptual model and seven research hypotheses were proposed. The empirical data were provided by a cross-sectional investigation including 459 medical and nonmedical employees of Romanian heath organizations, selected by a mixed method sampling procedure. A partial least squares structural equation modeling (PLS-SEM) approach was selected to provide information on the relevance and significance of the first- and second-order constructs, test the hypotheses, and conduct an importance performance matrix analysis. The PLS-SEM estimation showed positive and significant relationships between KM process and quality of healthcare, and organizational-level social and economic outcomes. Moreover, the research results provided evidences for the complex complementary mediation of the quality of healthcare and social-related outcomes on the relationships between KM process and social and economic outcomes. The theoretical and managerial implications are discussed and suggestions for future research are provided at the end of the paper.

## 1. Introduction

Given the rapid expansion of the global knowledge-based economy, organizations are facing a pressing need to identify and operationalize the most appropriate solutions that enable them to achieve a sustainable competitive advantage. Within the new knowledge-based economy, bases and banks of information and knowledge are created, managed within organizations, administrations, countries, and groups of countries, being valued as real “treasures” [1]. In the knowledge-based view, knowledge is considered a strategic asset in achieving a long-term competitive advantage. However, knowledge-based organizations must not only “treasure” a certain amount of knowledge, but should deliberately and systematically design the right processes through which new knowledge is acquired and ensure existing knowledge assets effectively contribute to the superior outcomes [2], encourage the best practices, and support continuous organizational learning [3].

Knowledge management (KM) is largely regarded as a dynamic and continuous process, involving various subprocesses and activities (i.e., creation/acquisition, storage, sharing, transfer, or utilization/application) [2,4,5]. It links operational management to strategic management [6], enabling individuals, teams, and communities to achieve superior performance by strengthening and supporting intellectual capital assets and their efficient use to deliver added value. Knowledge-based organizations, through the KM processes and practices they adopt, integrate KM into information systems and business processes at a higher level [6].

Despite the increasing emphasis placed on KM in the business sector, and the increasing amounts of literature dedicated to studying the particularities of this process (including its impact on different organizational outcomes), for the healthcare sector and health organizations, as stated by Van Beveren [7], few studies have investigated the specific aspects of KM and suggested outcomes that should be considered. In addition to the theoretical approaches, only a few descriptive qualitative case studies [8,9,10,11,12] and systematic reviews of existing studies [13,14,15,16] have been published, whereas quantitative research is almost nonexistent [17,18,19,20].

Employees of health organizations, especially the medical staff, are currently operating with a considerable amount of knowledge, while medical practice assumes operative knowledge retrieval for individual and team actions and decisions [10], as well as superior valorization. However, the implementation within the Romanian sanitary units of specific KM processes and practices, while creating the “knowledge-based healthcare organization” [12] is far from its potential. Many KM projects are information management projects aimed to handle data and information, without any contribution to processes and services innovation [21].

The existing theoretical framework, which helped to shape an overall picture of the KM process in the context of health organizations, as well as the issues that have not yet been sufficiently explained, led to the following questions that we will seek to find relevant answers through our research.

RQ1. What is the impact of the KM process on organizational-level outcomes in the healthcare context?
RQ2. What is the mechanism through which the KM process is able to enhance the quality of care and social and economic outcomes of health organizations?

Thus, the purpose of this paper was to provide a new perspective on the contribution of KM process (including knowledge acquisition, knowledge sharing, and knowledge utilization) to the healthcare quality improvements, as well as the social- and economic-related outcomes in the context of health organizations. For this purpose, building on the existing theoretical and empirical studies and our own previous research, an integrated model is proposed and was tested empirically. The model is intended to enrich the theoretical and empirical approaches to specific aspects of the KM process within health organizations, which, in turn, may support managerial practice in the operationalization of the most effective KM practices, leveraging the potential of the intellectual capital in enhancing the social and economic outcomes of health organizations.

The study proceeds as follows. Section 2 provides the theoretical background, Section 3 presents the development of the research hypotheses and the conceptual model, Section 4 provides our research methodology, Section 5 presents the results and analyses of the empirical data as well as the research hypotheses testing, and Section 6 is dedicated to discussion and conclusions, bringing together the theoretical and practical implications, along with research limitations and suggestions for future research.

## 2. Theoretical and Empirical Background

### 2.1. Knowledge Management Process and Organizational Performance

Organizations are starting to understand the importance of knowledge and KM in fostering organizational performance and enchanting their capacity to adapt and compete. Coupled to this understanding, researchers have become increasingly interested in developing theories, models, and tools through which they can better analyze and explain the KM process [7] and its underlying mechanisms that lead to enhanced performance. Bolisani and Bratianu [6] emphasized that the study of KM processes has no intrinsic value from either a theoretical or empirical point of view. KM only became important due to its ability to improve the quality of work within organization, thereby affecting organizational performance.

Grover and Davenport [22] identified two major streams in studying KM in the organizational field [22]. (1) The theoretical approach aims to explain the differences in firm performance from two perspectives: a knowledge-based view, which emphasizes the contribution of heterogeneous stocks and flows of knowledge to firm success and examining how the mechanism of integrating knowledge provide flexible capabilities. (2) The empirical-based stream of studies is mostly interested in the flows of knowledge and the determining factors of knowledge flows between organizations and organizations subsidiaries, emphasizing knowledge sharing from the perspective of interorganizational collaboration. 

Despite the later emphases on the KM concept and the common belief that KM positively contributes to organizational-level outcomes [23], knowledge, and empirical evidence able to explain the underling mechanism are lacking. 

Notably, measuring KM is not straightforward. Knowledge management (KM) is largely regarded as a dynamic and continuous process, involving various subprocesses and activities (i.e., creation/acquisition, storage, sharing, transfer, or utilization/application) [2,4,5]. Different authors have identified different frameworks that capture key aspects and processes of KM: knowledge generation, sharing, and use [23]; knowledge generation, codification, transfer, and realization [22]; knowledge creation process, knowledge capture, knowledge storage, knowledge organization process, knowledge dissemination, and knowledge application [24]; accumulation, sharing, use, and ownership [25]; acquiring, converting, applying/using, and protecting [21]; and knowledge acquisition, knowledge storage, transfer, sharing, knowledge application, and knowledge creation [26]. Probably one of the most comprehensible overviews of basic knowledge processes was provided by Bolisani and Bratianu [6], who described knowledge acquisition and creation, knowledge loss, knowledge distribution and sharing, knowledge transformation, knowledge storage and retrieval, and knowledge use. Knowledge processes are difficult to capture and describe since they are equally recursive, expanding, and often discontinuous processes [22]. Moreover, the components of knowledge processes are not strictly delimited, not always occurring in a linear sequence and often concurrent with each other [4]. In measuring KM, one should also considers the nature of knowledge involved: tacit knowledge or explicit knowledge [27]. Tacit knowledge is captured within individual skills or know-how and can sometimes be difficult to express in words, while explicit knowledge can be easily shared [28]. Nonaka [27] assumed that knowledge is created by conversion between tacit and explicit knowledge trough four basic processes/models: socialization, internalization, externalization, and combinations of these.

While organizations focus on knowledge as their essential assets, within this new knowledge paradigm, it appears the need to find new ways to measure organizational performance [29]. Measuring organizational performance in relation to KM processes plays a central role in the evaluation of knowledge strategy effectiveness, allowing academics and managers to identify critical areas and provide theoretical and empirical evidence to support continued improvements. Although the importance of measuring performance and its relationship with the KM process is widely accepted in academic and business environment, there is still a lack of consensus on the most appropriate models and methods [26] able to describe and explain this relationship.

Empirical studies have focused on the role of KM in different types of organizations and different organizational outcomes, which may be categorized into three main clusters: (1) managerial outcomes, such as operational performance [30], organizational effectiveness [21,23], and organizational performance (represented by quality, product and service innovation, and operating efficiency) [31]; (2) social outcomes, which may include employee satisfaction [32,33] and client satisfaction [26,31,34]; (3) financial (economic) outcomes including productivity [34], financial performance [26,31,35], and competitiveness [35]; and (4) measuring organizational performance across multiple interrelated perspectives [36,37,38,39,40,41] or interorganizational pathways [42], thus developing organizational performance assessment frameworks and models. For instance, Orzano et al. [36] considered organizational performance based on quality, products/services, productivity, and workplace and patient satisfaction; whereas other authors [26,43,44] proposed a balanced scorecard (BSC) perspective. Lyu et al. [26] introduced a framework for evaluating KM performance, linking specific KM processes with BSC outcomes, which incorporate four perspectives: financial, clients, internal business processes, and learning and growth, through a fuzzy evaluation method. In their conceptual model, knowledge creation is associated with clients’ perspectives; learning and growth; knowledge acquisition, storage, and transfer; and sharing with internal business processes. Knowledge application is associated with all four aspects of organizational outcomes. In turn, those different outcomes may be measured using different instruments.

A direct relationship between KM processes and organizational level outcomes has not been examined, as explaining organizational outcomes through only a single factor is insufficient and risky [4]. In order to understand the impact of KM processes on organizational performance, researchers have adopted two main strategies. The first approach involves considering KM process as an enabling mediator in the relationship between organizational performance and organization resources and capabilities, such as strategy, structure and culture [21,23], transformational leadership and quality management [18], self-learning and organizational learning [45], information technology [25], culture (collaboration, trust, and learning), structure (centralization and formalization), T-shaped skills, and information technology (IT) support [4]. Thus, Zheng et al. [23] suggested that KM should not only be considered an independent managerial practice, but also an intervening mechanism. They found that KM processes positively influence organizational effectiveness only if aligned with the organizational context (i.e., organizational structure, culture, and strategy). A second group of studies introduced intermediate outcomes, such as trust [46], organizational creativity [4], knowledge satisfaction [21] or decision-making (sense-making), and organizational learning [36], which help explain role of KM in creating organizational-level value, or argue for a moderating role of the context in which KM practices are used (e.g., nature of the tasks performed) [47].

### 2.2. Healthcare Perspective on Knowledge Management

Healthcare organizations have embraced the concept of KM after organizations within the business sector. KM began to penetrate the organizational and managerial processes in health organizations [13]. With respect to the healthcare sector, the topics debated in the literature may be grouped into three main streams [13]: (1) the nature of the knowledge concept in the healthcare context, along with its managerial consequences; (2) the potential advantages and pitfalls of specific KM initiatives and tools; and (3) enablers and barriers encountered by KM within healthcare organizations.

In terms of nature of knowledge relevant for health organizations, there are two types [27,28]: tacit knowledge and explicit knowledge. While explicit knowledge may be easily shared through various supports (e.g., individual electronic medical records, and databases or systems), tacit knowledge underlies personal skills and may be shared only within collaborative teams, direct interactions with other health professional or patients, or after conversion in explicit knowledge [13,27,48]. The tacit knowledge is particularly important for healthcare professionals considering the nature of their work and their professional culture, traditionally IT adverse [13]. Within the healthcare context, different “actors”/stakeholders should also be considered when analyzing KM. For instance, different kind of knowledge assets, processes, and outcomes may be relevant for medical staff, nonmedical employees of health organizations, patients, or the health organization.

Knowledge management (KM) is largely regarded as a dynamic and continuous process, involving various alternative and (sometime) concurrent subprocesses and activities (i.e., creation/acquisition, storage, sharing, transfer, or utilization/application) [2,4,5]. Different authors have identified different frameworks that capture key aspects and processes of KM. For the purpose of this paper, knowledge acquisition, knowledge sharing, and knowledge utilization were considered. Knowledge acquisition increase the “stock” of knowledge within organization generated inside organization or acquired from outside its boundaries [6]. To the traditional knowledge acquisition channels, such participation to medical congresses, seminars, and lecturing specialized publications, one may consider the electronic databases and medical electronic records [9,49].

Knowledge sharing is meant to restructure and increase the “stock” of knowledge by linking the individual level (where the knowledge lies) and organizational levels (where the knowledge is used). It is highly dependent on the willingness to share [6] and, therefore, on interpersonal and organizational factors. Within the healthcare context, interorganizational cooperation and collaboration [50] and involvement in social learning practices, such as communities of practice and professional networks [50], may enhance the knowledge sharing process. Sometimes, informal contexts, such as coffee breaks or informal meetings are preferred for sharing professional knowledge [9]. Nicolini [13] identified also a number of barrier in knowledge sharing in professional boundaries (between healthcare professionals and managers and between researcher and practitioners).

Knowledge use/utilization is the final stage and the purpose of the KM process [6] and is embedded within the very healthcare services. Knowledge use may be facilitated by clinical decision support systems [9,49].

The processes of creation, sharing, storage, and use/reuse of knowledge are especially challenging in health organizations due to the nature of knowledge, manifested through [50]: complexity of the system involved, the impact on medical errors, the increasing amount of knowledge involved in medical practice, and the high costs of providing healthcare.

Several main benefits of KM adoption were identified in healthcare organizations that may positively impact the individual practitioners and organizational outcomes [9,36,50]: (1) reduction of medical errors, (2) encouraging and supporting intra- and interorganizational cooperation and collaboration, (3) enhancing the overall quality of care, (4) cost reduction, (5) decision making/sense making through formalized decision procedures, and (6) organizational learning.

If the KM process has specific features and challenges for health organizations, the same can be said about organizational-level outcomes considered as performance indicators. As Van Beveren [7] stated, even if health organizations are motivated to address issues like cost control, quality of services, efficiency, and effectiveness, they are not primarily driven by increasing profit or market share. Healthcare organizations also face a shift from the central role of doctors and the quality of medical care, to a patient-centered process, concerned with the satisfaction of patient needs. In this context, Porter and Teisberg [51] introduced the concept of the “value for patient”, defined as the health outcomes that matter to patients in relation to the cost of achieving these results [52] or the perceived patient satisfaction due to medical service received relative to the price paid [53].

Cowing et al. [54] proposed a healthcare delivery triad to describe the performance of healthcare organizations from the perspective of three key players, each of them having a unique but interrelated perspective on healthcare organization performance: (1) the healthcare organization perspective, in terms of operational efficiency (including costs, times, and rates of service) and effectiveness (measured as clinical performance); (2) the clinician perspective, including needs related to job satisfaction and organizational support; and (3) the patient perspective, interested in subjective patient satisfaction perception of the quality of care, interpersonal relationships, meeting psychosocial needs, and the overall health outcome. There is evidence that KM strategies adopted within the healthcare sector benefit employees, patients, healthcare organizations, and overall public health [14].

## 3. Research Hypotheses and Conceptual Model

**Figure 1:**
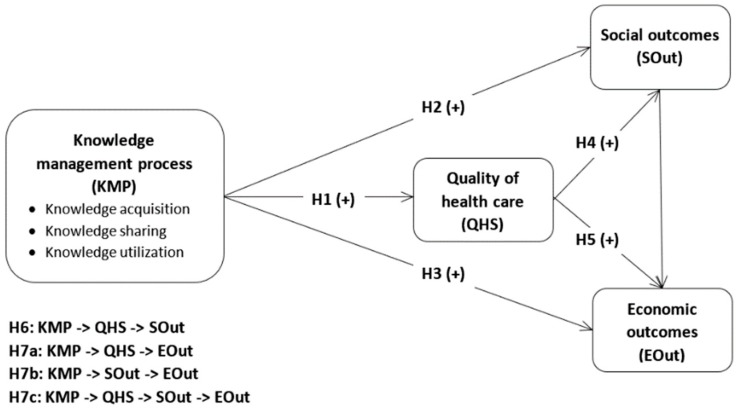
Conceptual model and hypotheses.

Given the above theoretical approach, past research, and our own experience, a conceptual model is proposed, categorizing three KM processes (knowledge acquisition, knowledge sharing, and knowledge utilization), quality of healthcare, social outcomes, and economic outcomes. Seven latent constructs, their hypothesized relationships, and subsequent rationales are illustrated in the conceptual model presented in Figure 1.

We assumed that KM processes (including knowledge acquisition, knowledge sharing, and knowledge utilization) are positively related to the quality of healthcare and the social and economic outcomes of a health organization (H1–H3). We also predicted that the quality of healthcare is positively associated with social and economic outcomes (H4 and H5). However, the positive relationships between KM process and organizational level social outcomes is mediated by the quality of health services (H6). We also assumed that a complex concurrent mediation process occurs within the relationship between KM process and economic outcomes: (1) KM process will positively impact the quality of care, which, in turn, will positively impact economic outcomes (H7a); (2) KM process will positively impact the social outcomes, which, in turn, will positively impact economic outcomes (H7b); and (3) quality of healthcare and social outcomes will sequentially mediate the positive relationship between KM process and economic outcomes (H7c).

## 4. Materials and Methods

### 4.1. Participants and Procedure

The empirical data for this research were provided by a cross-sectional investigation attended by employees of Romanian heath organizations, conducted at the end of 2017. As the research is addressed to a rare and specialized population (the employees of the healthcare system) and the studied phenomenon (knowledge management process) is manifested at the organizational level, a mixed method was chosen for the selection of the respondents. In the first step, a relevant number of health organizations were identified, with respondents being selected and participating in the survey in their own organizational environment. Within health organizations, the basic processes are those of providing medical care, and KM practices embedded within those processes are more likely to be translated into enhanced quality of care, patient satisfaction, health status, and life quality. The processes of acquiring, sharing, and using knowledge also involve auxiliary and managerial processes. For this reason, we considered the nonmedical employees of health organizations as necessary to include within the study sample (alongside the medical staff).

After checking for unusable responses, the remaining 459 questionnaires were further processed and analyzed. To capture the process of KM within health organizations as accurately as possible, the study sample included both medical (79.3%) and nonmedical staff (20.7%), occupying managerial (14.4%) and nonmanagerial positions (85.6%), with an average seniority within organization of 5.5 years. A complete picture of the respondents’ characteristics is presented in Table 1.

### 4.2. Measures

The questionnaire was designed using a multistage process. In the first step, as presented in the previous section, relevant literature was reviewed in order to identify the most suitable KM processes and outcomes to be considered in the health organization context as independent and dependent variables. The items included in the questionnaire (Appendix A) were inspired by the theoretical and empirical approaches of the analyzed concepts and previous research and were revised and refined through an iterative process.

In the first part of the questionnaire, the participants were informed of the aim of the study, that their participation is anonymous, and that their answers will be used only for scientific purposes. The final research instrument items were designed to measure six first-order and one second-order (hierarchical) latent constructs. (1) To measure the overall KM process, respondents were asked to what extent they agree with 17 statements related to knowledge acquisition, knowledge sharing, and knowledge use processes [18,21,25,30,34]. (2) Quality of healthcare was described by eight items corresponding to the dimensions of healthcare quality: professional competence, accessibility, interpersonal relationships, continuity of healthcare, efficiency, effectiveness, safety of care, and freedom of choice [56,57,58]. (3) The social outcomes construct emphasizes the performance of health organizations in terms of its most important stakeholders (patients and employees): employee satisfaction (four items), patient satisfaction (three items), patient quality of life (one item), and patient health status (one item) [51,59,60,61]. (4) The economic outcomes construct included three items measuring the level of competitiveness and three items for economic performance [60,61]. All items were measured on a five-point Likert scale (with the options ranging from “strongly disagree” to “strongly agree”), except for the economic performance and competitiveness items, measured also on a five-point scale, but the options ranged from “much worse” to “much better”. In addition, we collected demographic information on respondents’ characteristics at the professional and organizational levels.

### 4.3. Data Screening

Data collection using questionnaires is typically associated with several issues that potentially negatively impact the structural equation modeling (SEM) results. These typical issues, including missing data, outliers, suspicious response patterns, and data distribution [62], were properly addressed in this study. Since all questionnaire data were self-reported, common method bias potentially occurred, inflating the relationships between variables. Hence, Harman’s single factor test was employed to all variables subjected to an Exploratory Factor Analysis (EFA). The first unrotated factor we extracted accounted for 29.4% of the total variance explained and the forced unrotated single factor explained 29.8% of the total variance of the independent and dependent variables, indicating that the PLS-SEM results were not affected by common method bias.

### 4.4. Data Analysis

The PLS-SEM technique was selected to conduct the analysis in this study. Due to its ability to focus on the analysis of key sources of explanation for a certain target construct, PLS-SEM is primarily used in exploratory research and theory development [62,63,64].

A three-step approach was selected to examine and analyze the PLS-SEM model [65,66,67]: (1) measurement (outer) model assessment, (2) structural (inner) model assessment (including hypotheses testing), and (3) Importance Performance (Map) Matrix Analysis (IPMA).

The outer model relates the individual indicator variable to their respective constructs. In this study, the conceptual model was operationalized by six first-order reflective constructs (KAch: Knowledge acquisition; KSha: Knowledge sharing; KUtil: Knowledge utilization; QHS: Quality of health services; SOut: Social-related outcomes; and EOut: Economic-related outcomes), measured by multiple items. To build the second-order (hierarchical) construct of the KM process (KMP), based on three of the first-order constructs (KAch, KSha, and KUtil), repeated use of the manifest variables of the lower-order latent variables (indicator reuse technique) [68,69] was adopted. The measurement model was created to explain the relationships between indicator variables and the latent constructs they compose, but also between the second-order and the corresponding first-order constructs.

Once the measurement model was found reliable and valid, the next step was the assessment of the inner model, which relates the target-dependent constructs (i.e., QHS, SOut, and EOut) to other constructs. The structural model assessment is concerned with its predictive capability and interconstruct relationships [62], which enables testing complex hypothesized relationships [70].

Next was the Importance-Performance Matrix (Map) Analysis (IPMA). To extend the analysis of the direct and indirect relationships among the latent constructs represented in the structural model and gain more insights (from the managerial point of view) into the most efficient area for improvement of each target construct, IPMA [62,63,71] was employed. For each target construct, the matrix identifies the direct and indirect antecedent variables with high importance and relatively low performance. Thus, the managerial efforts directed at improving the performance of the associated area will more likely result in the enhanced performance of the target construct. To identify the specific areas for improvement, IPMA was conducted at the indicator level. The impact of a specific indicator on the target construct may be interpreted similarly to regression analysis: increasing the performance of a specific indicator would increase the performance of the target construct by the size of its unstandardized total effect (importance) [63].

In this study, two statistical packages were employed to implement the data analysis: IBM SPSS Statistics version 25.0 (IBM Corp., Armonk, NY, USA) [55] and SmartPLS version 3.2.7 (GmbH, Bönningstedt, Germany) [72].

## 5. Results

### 5.1. Measurement Model Assessment

Confirmatory factor analysis (CFA) was conducted to validate the measurement model in terms of indicator reliability, convergent validity, and internal consistency reliability. To assess indicator reliability, first, outer loadings were examined and three indicators with loadings lower than 0.5 were removed [65,73] (QHS1, QHS2, and LQual1). In addition, convergent validity was examined through the average variance extracted (AVE). In our measurement model, all AVE are above 0.5 [74], meaning that all latent constructs are able to explain most of the variance of their indicators. In terms of internal consistency reliability, three indicators were evaluated. The traditional Cronbach’s alpha and the alternative measure Rho_A both exceeded the established threshold value of 0.7 [75] for all latent constructs. The Composite Reliability (CR) value was calculated and found to be greater than 0.7 [74,76]. As shown in Table 2, all the indicators presented above support the indicator reliability, convergent validity, and internal consistency reliability of the measurement model.

Evaluation of the reflective first- and second-order constructs of the measurement model also included the discriminant validity. First, in examining the outer loading matrix, we found that each indicator loads higher on its construct than all the other constructs. According to previous research [74,77], each squared roots of the AVE of the latent variables (represented on the diagonal of the correlation matrix) was greater than the squared correlations of that variable to all other latent variables. This means that the all reflective constructs of the model were different from each other, and capture the meaning not represented by other constructs [62], thus supporting the discriminant validity of our model (Table 3).

### 5.2. Structural Model

To evaluate the structural model, collinearity issues, significance, and relevance of the model paths, coefficients of determination (*R*^2^) and the effect size (*f*^2^) were assessed. In the first step, to eliminate any suspicion of collinearity issues, variance inflation factors (VIF) and tolerance for each set of predictors were examined and their values were found below the recommended thresholds of 5 and 0.2, respectively [62].

The *R*^2^ values were calculated for all endogenous constructs to evaluate the predictive power of the structural model. As shown in Table 4, all variables representing the KM process are able to explain only 16.7% of the variance within the quality of health services (*R*^2^_QHS_ = 0.164). The rest of the 83.6% remaining can be explained by other variables not included in the model. However, KM processes and quality of health services together are better predictors of social-related outputs, since they explain the majority of variance (*R*^2^_SOut_ = 0.511), whereas KM processes, quality of health services (KHS), and social-related outputs explain 47.9% of the variance of economic-related outputs (*R*^2^_EOut_ = 0.479).

In addition to the *R*^2^ values, the effect size (*f*^2^) of the variables of interest was investigated to determine their impact on the endogenous variables if omitted from the model (Table 5). Considering established guidelines [62,78], the KM process has a medium effect on quality of healthcare, an almost large effect on social-related outcomes, and a small and not significant statistic effect on economic-related outcomes. In turn, the quality of health services has a large effect on social-related outcomes and a small and not significant effect on economic-related outcomes.

PLS-SEM does not emphasize the model fit, but instead maximizes the explained variance of the target constructs, considering this criterion as sufficient and meaningful fit evidence [71]. However, in this study, the overall model prediction performance (including measurement and the structural model) was evaluated using the global goodness of fit index (GOF). According to the Tenenhaus et al. [79] formula

(1) $$GOF=\sqrt{\bar{Comunality}\times \bar{R^{2}}}$$

and the guidelines introduced by Wetzels et al. [69] to evaluate the effect size of the GOF, the obtained value of GOF = 0.535 led us to conclude that the goodness of fit index is large enough to support the global model validity.

### 5.3. Testing Research Hypotheses

**Figure 2:**
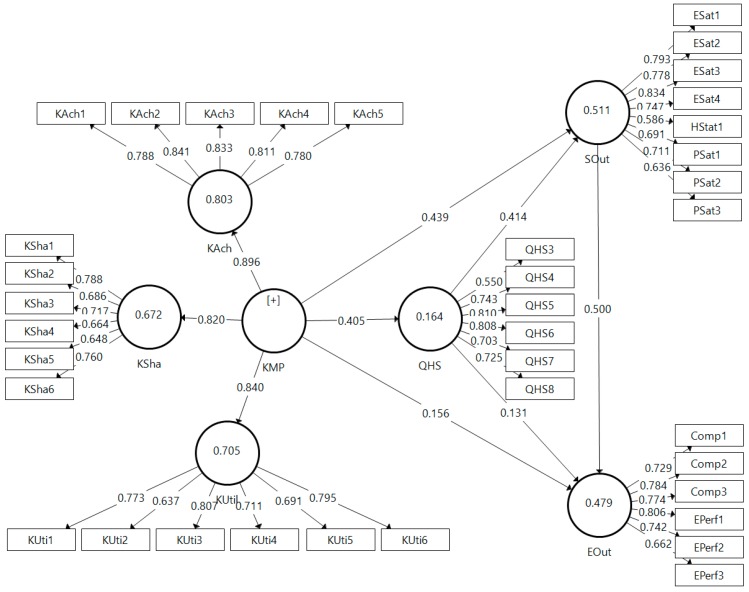
Measurement model computed with SmartPLS 3.2.7 (GmbH, Bönningstedt, Germany) [72]. Note: KAch—knowledge acquisition; KSha—knowledge sharing; KUtil—knowledge utilization; KMP—knowledge management process; QHS—Quality of health services; SOut—social-related outcomes; EOut—economic-related outcomes.

To evaluate the significance and relevance of the structural relationships between the latent constructs of the model, path coefficients were examined, along with their associated t-statistics and variance explained. By means of a bootstrapping procedure with 5000 resamples, the path significance of all hypothesized direct and indirect (total and specific) effects, as well as their bias-corrected confidence intervals, were computed (Figure 2).

The first three hypotheses (H1–H3) predicted that the KM process would significantly and positively influence quality of healthcare and organizational-level social and economic outcomes. As predicted, the findings in Table 5 and Figure 2 confirm that the KM process (represented by the second-order reflective construct KMP) significantly influences quality of healthcare (β_KMP→QHS_ = 0.407, *t* = 10.149, *p* < 0.001), social outcomes (β_KMP→SOut_ = 0.439, *t* = 14.747, *p* < 0.001), and economic outcomes (β_KMP→EOut_ = 0.156, *t* = 3.332, *p* < 0.01). Hence, H1, H2, and H3 were robustly supported. The direct and positive effects of the quality of healthcare on social and economic outcomes (β_QHC→SOut_ = 0.414, *t* = 10.817, *p* < 0.001; *β*_QHC→EOut_ = 0.131, *t* = 9.278, *p* < 0.01) confirm H4 and H5.

Hypothesis 6 stated that quality of healthcare mediates the positive relationship between knowledge process and social outputs. As shown in Table 6, the positive and statistically significant direct effect (*β*_KMP→SOut_ = 0.439, *t* = 14747, *p* < 0.001) and the positive and statistically significant indirect effect (*β*_KMP→QHS→SOut_ = 0.168, *t* = 7.390, *p* < 0.001) support the complementary mediation effect [62] of the quality of healthcare on the relationship between KM and social outputs.

Hypotheses 7a–7c assume that the relationship between KM process and economic outcome is mediated by quality of health services and social outcomes. As shown in Figure 2, in this situation, the mediation process is quite complex, since there are three concurrent specific indirect effects. Examining the direct effect between KM process and economic outcomes (*β*_KMP→EOut_ = 0.156, *t* = 3.332, *p* < 0.01) and the total indirect effect (β_KMP→EOut_ = 0.358, *t* = 12.888, *p* < 0.001), both are positive and statistically significant, suggesting a complementary mediation. However, to validate hypotheses 7a–7c, following the recommendations of Nitzi et al. [80] and Castro et al. [81], the specific indirect effects, associated *t*-statistics, and bias-corrected confidence intervals (computed with a bootstrapping procedure with 5000 resamples) were examined for each mediator and combination of mediators. Thus, the positive and significant specific indirect effect of quality of health services (*β*_KMP→QHS→EOut_ = 0.053, *t* = 2.585, *p* < 0.05), social outcomes (β_KMP→SOut→EOut_ = 0.220, *t* = 8.069, *p* < 0.001), and the combined specific indirect effect of the two mediators (quality of health services and social outcomes) (*β*_KMP→QHS→SOut→EOut_ = 0.084, *t* = 5.625, *p* < 0.001) support H7a, H7b, and H7c.

### 5.4. Importance Performance Matrix Analysis

**Figure 3:**
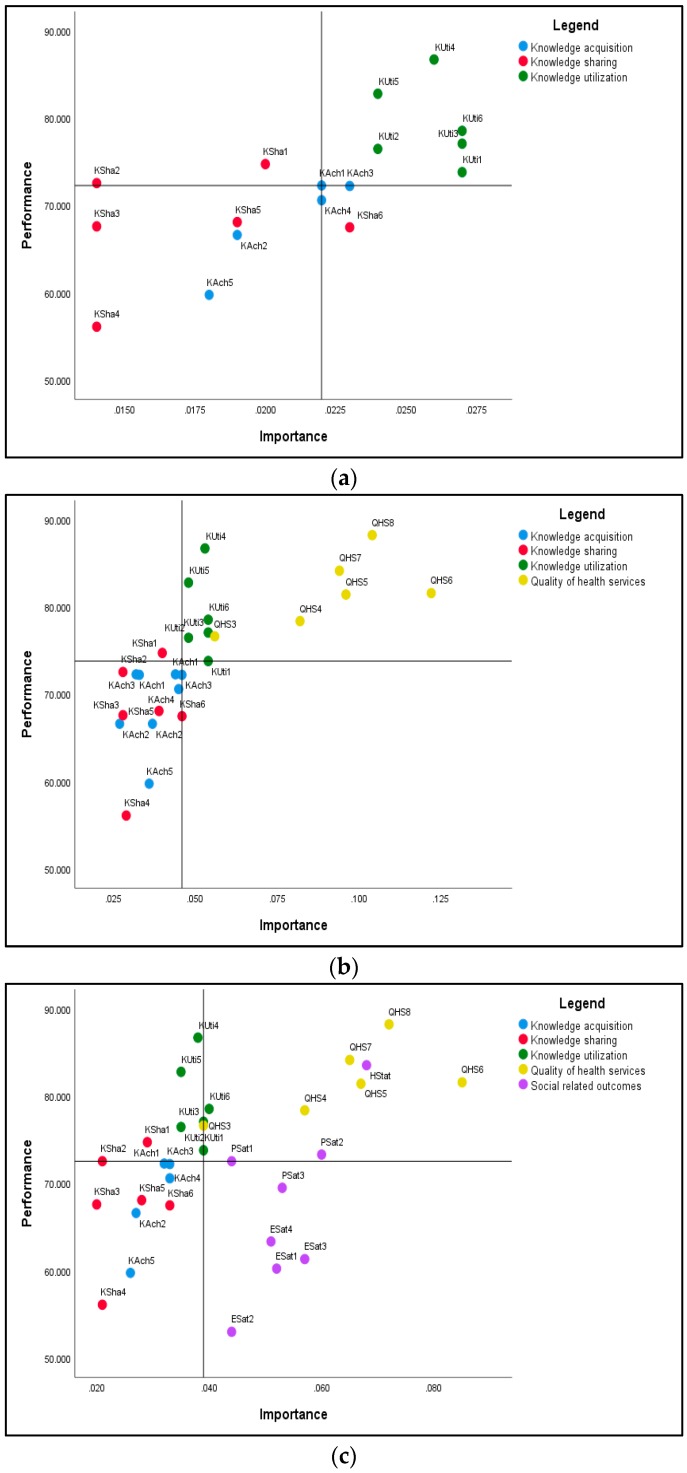
Importance-Performance Matrix Analysis (IPMA) for the target constructs: (**a**) quality of healthcare, (**b**) social outcomes, and (**c**) economic outcomes. Source: authors computation with SmartPls 3.2.7 (GmbH, Bönningstedt, Germany) [72] and IBM SPSS 25.0 (IBM Corp., Armonk, NY, USA) [55].

Figure 3 illustrates the IPMA results for the three target constructs: quality of healthcare (Figure 3a), social-related outcomes (Figure 3b), and economic outcomes (Figure 3c).

As shown in Figure 3, the knowledge use indicators are the most important for quality of care (Figure 3a), but also have the highest performance. The quality of healthcare indicators are the most important in terms of social outcomes (Figure 3b). Notably, for these two criteria, the indicators with relative high importance are also the high performing indicators. However, considering the economic outcomes criteria, the social outcomes indicators display a relatively medium importance and low-to-medium performance, whereas quality of care indicators are relatively medium-to-highly important and high performing (Figure 3c).

## 6. Discussion and Conclusions

### 6.1. Theoretical and Practical Implications

This study aimed to propose a conceptual model and provide empirical evidences to explain the contribution of KM process to organizational-level social and economic outcomes in the context of health organizations. The mediating role of quality of healthcare and social outcomes were also considered. In the first step, based on the theoretical background and our own research, a conceptual model was proposed, illustrating the relationships between six first-order and one second-order latent constructs. We proposed seven research hypotheses. To verify the relationships between latent constructs, partial least squares structural equations analysis modeling (PLS-SEM) was performed using SmartPLS version 3.2.7 (GmbH, Bönningstedt, Germany) [72] statistical software.

The overall results support the positive impact of the second-order reflective construct of KM process on social and economic outcomes, but those relationships are partially mediated by the quality of healthcare and the social outcomes. As such, the results have theoretical and managerial implications. The IPMA produced more insights into the underlying mechanism that links KM process and organizational level outcomes by identifying (for each target outcome) the area of managerial interventions that would more likely result in enhanced outcomes.

As previously stated, the KM process has no intrinsic value, only becoming important through its ability to foster the quality of other processes within an organization, thereby enabling organizational performance [6]. Therefore, we concentrated on discussing the effect of KM on each of the outcomes of health organization considered in this research, highlighting both the theoretical and practical implications.

#### 6.1.1. Quality of Healthcare

In this study, the quality of care was considered both a goal whose level is influenced by the KM process, as well as a mediating factor between the KM process and social and economic results. As a distinct outcome, quality of care is positively influenced by the KM process. Even though this relationship is direct within the model, the mechanism that enables it is quite complex, especially in the context of health services [9,36,50]. In terms of direct effect, KM processes significantly influences quality of healthcare. Beside the direct effect, should be also discussed the effect size (*f*^2^) (i.e., the impact on the endogenous variables if omitted from the model). The research results outlined a medium effect of knowledge management processes on quality of care (*f*^2^ = 0.203, *p* < 0.001), which, for instance, is higher than on economic outcomes. This effect confirms the initial assumption (hypothesis 1) and previous theoretical and empirical studies [9,36,50], and will be discussed below.

Thus, a first mentioned benefit of the KM process is the reduction of medical errors by providing decision support tools based on rules and established reasoning. Medical errors reduction may have positive consequences in terms of quality of care, but also on patient satisfaction and costs involved in healthcare [50] on the other two considered outcomes (social and economic).

The KM process also encourages and supports intra- and interorganizational cooperation and collaboration, which are vital factors in avoiding errors and delivering quality of care [50]. Knowledge acquisition and sharing, by involvement in social learning practices such as communities of practice and professional networks and knowledge assimilation and application, and facilitated by the adoption of clinical decision support systems, may have implications on the interpersonal and technical performance of healthcare delivery [9], and therefore on employee and patient satisfaction and the overall health benefits of patients.

In terms of decision-making/sense-making, healthcare delivery currently involves a considerable amount of information and knowledge, which is not always properly systematized, accessible, or in the best form. In this context, a formalized decision process helps make sense of knowledge, encouraging new knowledge production, access and sharing, and supporting collective actions [36].

Decision making methods positively influence the organizational learning, but equally, organizational learning may enhance the decision-making process [36]. Decision-making (sense-making) and organizational learning help explain the enabling role of finding, sharing, and developing knowledge in creating organizational-level value [36].

Finally, the KM is able to enhance the overall quality of care. Adoption of KM practices by finding, collaborating, sharing, and developing medical practitioner knowledge ultimately results in increased quality of care [50]. Capturing and sharing patient data, facilitated by the adoption of electronic medical records, positively impact healthcare delivery [9].

The practical implications of those findings lie in the possibilities outlined for managerial interventions in order to increase the quality of medical services by paying particular attention to the KM process. The IPMA provided highlighted the specific areas requiring intervention. Since the most important specific areas (the highest impact) on the quality of health services are those of knowledge use, the efforts directed toward knowledge use will have the greatest impact on the quality of healthcare. However, knowledge use is already medium and highly performing, leaving relatively little room for improvement. Therefore, in attempting to improve the quality of care, managers should direct their efforts to specific areas of knowledge use (those displaying medium performance) and selecting, acquiring, and sharing knowledge with medium impact on quality of care.

#### 6.1.2. Social Outcomes

For healthcare organizations, social outcomes, including employees’ satisfaction, patient satisfaction, and patient health status, are the reasons for their existence. Hence, the increased importance placed on identifying the most effective means to maximize this type of outcome. Focusing on the social outcomes, KM has a direct impact of KM on social outcomes (i.e., employee satisfaction, patient satisfaction, and patient health status), and a positive indirect impact, mediated by the quality of healthcare. In other words, the KM process positively impacts the quality of healthcare, which, in turn, has a positive effect on social outcomes. Beside the direct and indirect effects, should be also mentioned that KM processes have a higher effect on social outcomes (*f*^2^ = 0.334, *p* < 0.001), than on the other considered endogenous constructs (quality of care and economic outcome). These results are in line with specialized literature outlining the positive link between KM process and each of the considered social outcomes [8,14,32].

Furthermore, IPMA provides useful insights into the most efficient areas of intervention for increasing the social outcomes. Not surprisingly, the quality of healthcare has the highest total effect (importance) on the social outcomes, indicating that a one unit increase in their performance will lead to an increase in the performance of social outcomes equal to the unstandardized value of their total effect. However, since most of the quality of care indicators are already high performing, for improvement, managerial interventions should be selected only the dimensions of quality of care with medium performance, to which some elements of knowledge use could be selectively added.

#### 6.1.3. Economic Outcomes

Although the social outcomes should be considered of maximum importance for health organizations, the economic outcomes (i.e., economic performance and competitiveness) cannot be neglected, especially as the healthcare services market is becoming increasingly competitive. If we consider the economic results, the relationship between the two constructs is quite complex. Besides the positive impact, which is not very strong, a much stronger total indirect effect was observed due to a complex concurrent mediation process, exercised by the quality of healthcare services, social outcomes and, sequentially, by both of them together.

Although such a complex relationship was not mentioned in previous research, previous studies outlined the positive effect on KM on different economic outcomes of health organizations. As already discussed, knowledge sharing through collaborative networks and reduction of medical errors have a positive impact on costs involved in healthcare. Decision support tools may assist with effective and efficient medical decisions, with favorable consequences on cost reduction, but also the overall quality of care and patient satisfaction [50]. Moreover, there were mentioned applications of artificial intelligence (AI), within the context of KM, which may be beneficial in improving the health organizations and health system’s efficiency and costs reducing [82].

It also worth to be mentioned that the effect size (*f*^2^) of the KM processes on economic outcomes (*f*^2^ = 0.033, ns) is smaller than the other two considered endogenous constructs (quality of care and social-related outcomes). Those results may be explained by the primary focus of healthcare organizations to maximize the value for patient (including through the quality of care), not the economic outcomes.

Considering the above, it would be useful to identify specific areas of managerial intervention with the highest impact on economic outcome. In analyzing the IPMA build for the economic outcomes as the target construct, we noticed the relatively medium-to-high importance of quality of care indicators, but also their high performance. The social outcomes indicators displayed a relatively medium importance and low-to-medium performance. Thus, the managerial interventions should be directed to selected quality of care dimensions, and especially the employees and patient satisfaction aspects.

### 6.2. Limitations and Directions for Future Research

This study has some limitations, which provide opportunities for future lines of research. First, the study design only analyzed the relationships between the KM process and organizational-level outcomes in the healthcare organizations context, without considering KM enablers such as organizational structure, IT support, organizational culture, or strategy. Based on the findings of this study, future research may extend the conceptual model to include selected KM enablers and consider the relationships with the variables within the present model. Second, we did not consider the moderating effect of other variables, such as organizational characteristics (type of healthcare, organizations size, and age) or the task involved in the relationships between KM process and endogenous constructs. Moreover, the study analyzed KM processes only at organizational level, not considering the specificity of KM assets and subprocesses of each category of healthcare employees (i.e., medical and nonmedical). Including such moderators into the model represents another area of future research. Third, an assumed limit of the study is nonprobabilistic sampling procedure bias. In future research, a probabilistic sampling method would improve the generalizability of the research results.

## Figures and Tables

**Table 1:** Professional and organizational characteristics of the sample (*N* = 459).

Variable		*N*/Mean	SD (%)
Profession	Physician	99	21.569
	Pharmacist	115	25.054
	Medical staff with higher education (other than physician)	24	5.229
	Nurse	101	22.004
	Nonmedical staff	95	20.697
	Others	25	5.447
Managerial position	Yes	66	14.379
	No	393	85.621
Seniority within organization (years)		5.538	4.412
Type of healthcare	Specialized medical care	141	30.719
	Hospital	190	41.394
	Pharmacy	128	27.887
Organization size (employees)	<10	87	18.954
	10–49	40	8.715
	50–249	144	31.373
	>250	188	40.959

Source: authors computation with IBM SPSS 25.0 (IBM Corp., Armonk, NY, USA) [55].

**Table 2:** Construct reliability and validity.

Latent Construct (First-Order/Second-Order; Reflective/Formative)	Items	Loadings	*SD*	*t*	α	Rho_A	CR	AVE
KAch—Knowledge acquisition	KAch1	0.788 ***	0.019	40.497	0.870	0.871	0.906	0.658
(first-order; reflective)	KAch2	0.841 ***	0.016	53.782				
	KAch3	0.833 ***	0.018	47.596				
	KAch4	0.811 ***	0.016	50.852				
	KAch5	0.780 ***	0.021	37.084				
KSha—Knowledge sharing	KSha1	0.788 ***	0.021	37.647	0.805	0.816	0.860	0.507
(first-order; reflective)	KSha2	0.686 ***	0.031	22.183				
	KSha3	0.717 ***	0.026	27.842				
	KSha4	0.664 ***	0.034	19.548				
	KSha5	0.648 ***	0.033	19.528				
	KSha6	0.760 ***	0.021	35.571				
KUtil—Knowledge utilization	KUti1	0.773 ***	0.022	35.005	0.831	0.842	0.877	0.545
(first-order; reflective)	KUti2	0.637 ***	0.039	16.383				
	KUti3	0.807 ***	0.018	44.961				
	KUti4	0.711 ***	0.028	25.318				
	KUti5	0.691 ***	0.032	21.806				
	KUti6	0.795 ***	0.016	49.245				
QHS—Quality of health services	QHS3	0.550 ***	0.051	10.883	0.820	0.838	0.870	0.531
(first-order; reflective)	QHS4	0.743 ***	0.029	25.704				
	QHS5	0.810 ***	0.021	39.429				
	QHS6	0.808 ***	0.019	43.494				
	QHS7	0.703 ***	0.031	22.830				
	QHS8	0.725 ***	0.029	25.022				
SOut—Social-related outcomes	ESat1	0.794 ***	0.019	41.999	0.869	0.875	0.898	0.527
(first-order; reflective)	ESat2	0.779 ***	0.024	32.619				
	ESat3	0.835 ***	0.014	61.671				
	ESat4	0.746 ***	0.024	31.178				
	HStat1	0.588 ***	0.040	14.578				
	PSat1	0.690 ***	0.027	25.625				
	PSat2	0.709 ***	0.025	28.317				
	PSat3	0.634 ***	0.030	21.377				
EOut—Economic-related outcomes	Comp1	0.727 ***	0.022	32.810	0.845	0.849	0.885	0.564
(first-order; reflective)	Comp2	0.781 ***	0.020	39.030				
	Comp3	0.772 ***	0.023	33.777				
	EPerf1	0.808 ***	0.016	49.829				
	EPerf2	0.747 ***	0.023	32.156				
	EPerf3	0.662 ***	0.031	21.079				
KMP—Knowledge management process	KAch	0.896 ***	0.011	84.809	0.907	0.914	0.889	0.727
(second-order; reflective)	KSha	0.820 ***	0.021	39.973				
	KUtil	0.840 ***	0.014	58.747				

*** *p* < 0.001; SD, Standard deviation; α, Cronbach’s Alpha; AVE, Average variance extracted; and CR, Composite reliability. Source: computation with SmartPls 3.2.7 (GmbH, Bönningstedt, Germany) [72].

**Table 3:** Discriminant validity.

Construct	EOut	KAch	KSha	KUtil	QHS	SOut
EOut	**0.751**					
KAch	0.431	**0.811**				
KSha	0.318	0.676	**0.712**			
KUtil	0.528	0.606	0.493	**0.738**		
QHS	0.488	0.276	0.135	0.571	**0.728**	
SOut	0.671	0.518	0.36	0.631	0.592	**0.726**

Note: Computation with SmartPls 3.2.7 (GmbH, Bönningstedt, Germany) [72].

**Table 4:** Coefficient of determination (*R*^2^) values of the endogenous constructs and effect size (*f*^2^).

Endogenous Construct	*R* ^2^	Relationship	*f* ^2^	Decision
QHS	0.164 ***	KMP → QHS	0.203 ***	Medium
EOut	0.479 ***	KMP → EOut	0.033	Small
		QHS → EOut	0.024	Small
SOut	0.511 ***	KMP → SOut	0.334 ***	Large
		QHS → SOut	0.299 ***	Medium

Note: *** *p* < 0.001; ** *p* < 0.01; * *p* < 0.05. Source: computation with SmartPls 3.2.7 (GmbH, Bönningstedt, Germany) [72].

**Table 5:** Testing for direct effects.

Hypothesis	Relationship	β	SE	t	95% BC CI		Supported (Yes/No)
					CI_low_	CI_high_	
H1 (+)	KMP → QHS	0.407 ***	0.040	10.149	0.340	0.471	Yes
H2 (+)	KMP → SOut	0.439 ***	0.030	14.747	0.390	0.488	Yes
H3 (+)	KMP → EOut	0.156 **	0.047	3.332	0.079	0.233	Yes
H4 (+)	QHS → SOut	0.414 ***	0.038	10.817	0.349	0.476	Yes
H5 (+)	QHS → EOut	0.131 **	0.054	9.278	0.051	0.214	Yes

Note: *** *p* < 0.001; ** *p* < 0.01; * *p* < 0.05; β, Standard coefficients; SE, Standard error; BC CI, bias-corrected confidence intervals. Source: authors computation with SmartPls 3.2.7 (GmbH, Bönningstedt, Germany) [72].

**Table 6:** Testing for direct effects.

Hypothesis	Relationship (Effect Type)	β	SE	t	95% BC CI		Mediation	Supported (Yes/No)
					CI_low_	CI_high_		
H6	KMP → SOut (Direct Effect)	0.439 ***	0.030	14.747	0.390	0.488	Simple complementary mediation	Yes
	KMP → QHS → SOut (Indirect Effect)	0.168 ***	0.023	7.390	0.131	0.206		
	KMP → SOut (Total Effect)	0.607 ***	0.026	23.646	0.560	0.646		
H7a–H7c	KMP → EOut (Direct Effect)	0.156 **	0.047	3.332	0.079	0.233	Multiple complementary mediation	Yes
	KMP → EOut (Total Indirect Effect)	0.358 ***	0.028	12.888	0.309	0.400		
	KMP → QHS → EOut (Specific Indirect Effect)	0.053 *	0.020	2.585	0.022	0.090		
	KMP → SOut → EOut (Specific Indirect Effect)	0.220 ***	0.027	8.069	0.176	0.267		
	KMP → QHS → SOut → EOut (Specific Indirect Effect)	0.084 ***	0.015	5.625	0.062	0.111		
	KMP → EOut (Total effect)	0.513 ***	0.039	13.160	0.443	0.573		

Note: *** *p* < 0.001; ** *p* < 0.01; * *p* < 0.05. Source: authors computation with SmartPls 3.2.7 (GmbH, Bönningstedt, Germany) [72].

## Appendix A

**Table A1:** Conceptual framework of variables.

Concept	Variable	Item	References
**KM process**		Within the organization…	
Knowledge acquisition	KAch1	New sources of information and knowledge are constantly being identified	[18,21,30,34]
	KAch2	New information and knowledge are acquired through participation at medical conferences and congresses	
	KAch3	New information and knowledge are acquired by studying relevant literature	
	KAch4	New information and knowledge are acquired by attending training or specialization courses	
	KAch5	New information and knowledge are acquired from high-quality medical centers	
Knowledge sharing	KSha1	Information and knowledge are frequently shared with department colleagues	[21,25,30,34]
	KSha2	Information and knowledge are frequently shared with younger/less experienced colleagues	
	KSha3	Information and knowledge are frequently shared with colleagues from other departments	
	KSha4	Information and knowledge are frequently shared with colleagues from other health organizations	
	KSha5	Information and knowledge are frequently shared by means of formal communication (e.g., meetings)	
	KSha6	Information and knowledge are frequently shared by means of informal communication	
Knowledge utilization	KUti1	Information and knowledge are cherished for their true value	[21,25,34]
	KUti2	Information and knowledge are considered as the organization’s valuable assets	
	KUti3	Different sources of information and knowledge are effectively used within medical practice	
	KUti4	Medical staff knowledge is effectively applied within their medical practice	
	KUti5	In providing medical care, there are effectively used medical protocols, procedures, and instructions existing within the organization	
	KUti6	The information and knowledge existing within the organization are accessible to those who need it	
**Quality of health services**			
	QHS1 ^a^	The organization has a highly skilled medical staff	[56,57,58]
	QHS2 ^a^	The medical services provided are accessible in terms of location, price, and waiting time	
	QHS3	Patients positively appreciate the quality of medical services in terms of interpersonal relationships	
	QHS4	Continuity in medical care is ensured	
	QHS5	The medical services provided are efficient	
	QHS6	The medical services provided are considered effective by patients and healthcare professionals	
	QHS7	There are no risks associated with the process of granting medical care	
	QHS8	Patients are provided free choice in terms of medical care	
**Social-related outcomes**			
Employees satisfaction	ESat1	The organization frequently measures employee perception of motivating factors	[51,59,60,61]
	ESat2	The organization frequently measures employees’ perception of demotivating factors	
	ESat3	Employee satisfaction has, overall, an increasing tendency	
	ESat4	Employee satisfaction is, overall, superior to that recorded in similar organizations	
Health status and quality of life improvement	HStat1	Medical services provided contribute to improving patient health status	[51,61]
	LQual1 ^a^	Medical services provided contribute to increasing patient quality of life	
Patient satisfaction	PSat1	Patient satisfaction with medical services/products is constantly assessed through surveys	[51,59,60,61]
	PSat2	Patient satisfaction has, overall, an increasing tendency	
	PSat3	Patient satisfaction is, overall, superior to that recorded in similar organizations	
**Economic-related outcomes**			
Competitiveness	Comp1	Evaluation of organization’s competitiveness… … compared with that of the main competitors	[60,61]
	Comp2	… compared to five years ago	
	Comp3	… compared with its set objectives	
Economic performance	EPerf1	Evaluation of organization’s competitiveness… … compared with its set objectives	[60,61]
	EPerf2	… compared with that of the main competitors	
	EPerf3	… compared to five years ago	

Note: ^a^ The indicators were not included into the final model.
